# Desiccation Treatment and Endogenous IAA Levels Are Key Factors Influencing High Frequency Somatic Embryogenesis in *Cunninghamia lanceolata* (Lamb.) Hook

**DOI:** 10.3389/fpls.2017.02054

**Published:** 2017-12-05

**Authors:** Xiaohong Zhou, Renhua Zheng, Guangxin Liu, Yang Xu, Yanwei Zhou, Thomas Laux, Yan Zhen, Scott A. Harding, Jisen Shi, Jinhui Chen

**Affiliations:** ^1^Key Laboratory of Forest Genetics and Biotechnology, Ministry of Education, Nanjing Forestry University, Nanjing, China; ^2^Co-Innovation Center for Sustainable Forestry in Southern China, College of Forestry, Nanjing Forestry University, Nanjing, China; ^3^The Key Laboratory of Timber Forest Breeding and Cultivation for Mountainous Areas in Southern China, State Forestry Administration Engineering Research Center of Chinese Fir, Fujian Academy of Forestry, Fuzhou, China; ^4^Centre for Biological Signaling Studies, Faculty of Biology, Albert Ludwigs University of Freiburg, Freiburg, Germany; ^5^School of Forestry and Natural Resources, University of Georgia, Athens, GA, United States

**Keywords:** ABA, desiccation, endogenous hormone, PEG, somatic embryo, *SOMATIC EMBRYOGENESIS RECEPTOR KINASE* (*SERK*), *WUSCHEL* homeobox (*WOX*)

## Abstract

*Cunninghamia lanceolata* (Lamb.) Hook (Chinese fir) is an important tree, commercially and ecologically, in southern China. The traditional regenerating methods are based on organogenesis and cutting propagation. Here, we report the development of a high-frequency somatic embryogenesis (SE) regeneration system synchronized via a liquid culture from immature zygotic embryos. Following synchronization, PEM II cell aggregates were developmentally equivalent in appearance to cleaved zygotic embryos. Embryo and suspensor growth and subsequent occurrence of the apical and then the cotyledonary meristems were similar for zygotic and SE embryo development. However, SE proembryos exhibited a more reddish coloration than zygotic proembryos, and SE embryos were smaller than zygotic embryos. Mature somatic embryos gave rise to plantlets on hormone-free medium. For juvenile explants, low concentrations of endogenous indole-3-acetic acid in initial explants correlated with improved proembryogenic mass formation, and high SE competency. Analysis of karyotypes and microsatellites detected no major genetic variation in the plants regenerated via SE, and suggest a potential in the further development of this system as a reliable methodology for true-to-type seedling production. Treatment with polyethylene glycol (PEG) and abscisic acid (ABA) were of great importance to proembryo formation and complemented each other. ABA assisted the growth of embryonal masses, whereas PEG facilitated the organization of the proembryo-like structures. *SOMATIC EMBRYOGENESIS RECEPTOR KINASE SERK*) and the *WUSCHEL* homeobox (*WOX*) transcription factor served as molecular markers during early embryogenesis. Our results show that *ClSERKs* are conserved and redundantly expressed during SE. *SERK* and *WOX* transcript levels were highest during development of the proembryos and lowest in developed embryos. *ClWOX13* expression correlates with the critical transition from proembryogenic masses to proembryos. Both *SERK* and *WOX* expression reveal their applicability in Chinese fir as markers of early embryogenesis. Overall, the findings provided evidence for the potential of this system in high fidelity Chinese fir seedlings production. Also, SE modification strategies were demonstrated and could be applied in other conifer species on the basis of our hormonal, morphological and molecular analyses.

## Introduction

*Cunninghamia lanceolata* (Lamb.) Hook, Chinese fir, is an important native evergreen tree species in China. This fast-growing conifer has been cultivated for over 3,000 years due to its elite wood attributes and high timber productivity (Yang, 1998). Chinese fir is now the dominant tree species in southern China, growing in over 15 southern provinces. The total planted area of Chinese fir has reached 9 × 10^6^ hm^2^ and accounts for ∼30% of the forested land in China (Lu et al., 2015). Chinese fir occupies prominent roles in ecological and commercial prospects.

Advances in third generation recurrent genetic selection and hybridization of Chinese fir through conventional breeding programs have led to great genetic improvements on growth, wood qualities and biological or non-biological stress tolerances. However, conventional breeding improvements are inefficient, costly, and time consuming due to the inherently long life cycles, unstable maturation, and frequently unavoidable dilution of desirable traits caused by genetic segregation and gene flow (Cairney et al., 1999). As an alternative to conventional breeding, somatic embryogenesis (SE) is thought to be the most promising clonal propagation strategy based on recurrent genetic selection for commercial plantations regeneration. The generation circulation time could be reduced, and the extra genetic gain of forest trees can be captured through SE with lower risks and costs (Gupta and Grob, 1995). The process can be rigorously controlled, and is theoretically feasible for all plant species. A well-established SE line, combined with cryopreservation, would be an excellent platform for long-term conversation and large-scale planting materials production (Park, 2002).

Since embryo-like structures were induced *in vitro* into somatic embryos in *Pinus banksiana* (Durzan and Chalupa, 1976), much progress has been archived by SE systems in conifers (Klimaszewska and Cyr, 2002; Cairney and Pullman, 2007; Jain et al., 2013). However, most studies have concentrated on pine species (Klimaszewska and Cyr, 2002; Sutton, 2002). *In vitro* culture of Chinese fir is more difficult in comparison to the other coniferous species. At the same time, a rapid mass propagation system is required to meet the increasing demand for this species. To our knowledge, less progress has been achieved for *C. lanceolata* SE than for propagation via organogenesis (Zhu et al., 2007a,b; Zhou et al., 2013; Li et al., 2017). Development of SE has been handicapped by limited yields and calluses’ necrosis (Kang, 2008; Chen et al., 2017). Xi and Shi (2005, 2006) used explants of cotyledon, hypocotyl and mature zygotic embryos for direct SE induction, which is not perspective for mass propagation and further genetic improvements due to low multiplication rates. Hu et al. (2017) recently established SE via embryogenic callus using immature zygotic embryos. The system employed ABA and PEG to successfully produce early somatic embryos, but normally developed, late stage somatic embryos were not obtained, and there were no reports about the conversion from somatic embryos to plantlets.

Members of the *SOMATIC EMBRYOGENESIS RECEPTOR KINASE* (*SERK*) gene family encode leucine-rich repeat-containing transmembrane proteins that are involved in signal transduction and are strongly related to SE (Santos and Aragão, 2009). SERK was first discovered as a marker for the somatic to embryogenic transition in carrot (Schmidt et al., 1997). The *WUSCHEL* (*WUS*) homeobox (*WOX*) transcription factor is described as functioning in early embryo patterning and lateral organ development in Arabidopsis (Haecker et al., 2004). Overexpression of *WUS* can initiate acquisition of embryogenic competence in Arabidopsis (Zuo et al., 2002) and cotton (Zheng et al., 2014). In *Picea abies, PaWOX2* and *PaWOX8/9* were highly expressed in early stage embryo development (Palovaara and Hakman, 2008; Hedman et al., 2013), when the primary body axis and radial patterning was being established (von Arnold et al., 2016). Therefore, *SERK* and *WUS* prompted SE during early embryogenesis (Guan et al., 2016).

In this study, we developed an effective synchronized SE system from *C. lanceolata* immature seeds. Cell masses were synchronized in liquid suspension before somatic embryo differentiation. This system produces seedlings with no cytological variation detectable by karyotype and microsatellite analyses. We showed that the competence of *C. lanceolata* seeds to generate somatic embryos correlated with developmental stage exhibiting low endogenous indole-3-acetic acid (IAA) hormone levels in the source materials. We further demonstrated that morphological differences were caused by exposure to polyethylene glycol (PEG) and abscisic acid (ABA) during the early developmental stages of somatic embryos. In addition, expression profiles of *SERK* and *WUS* reveal their applicability as the markers of early embryogenesis of Chinese fir.

## Materials and Methods

### Plant Materials

Chinese fir cones were collected once a week from late June to late August 2009 in a National Clonal Seed Orchard at Yangkou and Shaowu Forest Farm, Fujian Province, China. These cones were immediately placed on ice and brought back to the lab within 24 h. Cones were stored at 4–5°C for no more than 1 week until further used.

Seeds with immature zygotic embryos of different developmental stages were used as the initial explants to induce somatic embryos. The stages of the zygotic embryo materials were identified from 5 to 6 randomly selected seeds of each cone according to Shi et al. (2010). Cones were opened and seeds were collected before sterilization. Seeds were then washed with detergent for 10 min, transferred into running water for 30 min, surface-sterilized with 75% (v/v) ethanol for 30 s, treated with 0.1% (w/v) HgCl_2_ containing a few drops (0.01%) of Tween 20 for 8–10 min, and then rinsed three times in sterile distilled water. Seed coats were removed as previously described (Becwar and Pullman, 1995; Pullman and Johnson, 2002), and the embryo sacs, including the zygotic embryos, were used for induction of proembryogenic masses (PEMs). More than 30 seeds of the same embryonic stage were used to assess the frequency of outgrowths and the test was repeated three times.

### Medium and Culture Conditions

The embryo sacs were cut near the suspensors and initially cultured for 1 month at 23–25°C in darkness on Gupta and Durzan medium (DCR) (Gupta and Durzan, 1985), which contained 2.0–6.0 mg L^-1^ 2,4-dichlorophenoxyacetic acid, 0.5 mg L^-1^ benzylaminopurine, 500 mg L^-1^ casein hydrolysate (CH) (Sigma), 450 mg L^-1^ L-glutamine, 100 mg L^-1^ myo-inositol, 20 g L^-1^ maltose, and 2.1 g L^-1^ gellan gum (Sigma). The medium was adjusted to pH 5.8 using KOH or HCl after the addition of all of the ingredients except the gelling agent. The gelling agent was added prior to autoclaving at 121°C for 20 min. Maltose was used as a carbon source and autoclaved separately.

For the synchronization of SE development, approximately 2 g of embryogenic calluses were suspended in 50 mL liquid medium consisting of DCR salts, 3 mg L^-1^ ABA (Sigma), 0.5 mg L^-1^ gibberellic acid (GA_3_), 500 mg L^-1^ CH, and 30 g L^-1^ maltose. ABA was filter-sterilized and added into autoclaved cooled medium.

After 3 weeks suspension culture, steadily proliferating cell clumps were dispersed on DCR solid medium containing 3 mg L^-1^ ABA, 1 mg L^-1^ GA_3_, and 120–200 g L^-1^ PEG (MW 8000; Amresco). The osmotic potential was measured by a Wescor 5520 vapor pressure osmometer (Wescor, Inc.). Three months later, the mature somatic embryos were transferred to basic DCR medium for the regeneration of plantlets, which were maintained at 25°C under cool white fluorescent light (30 μmol m^-2^ per second, with a 16-h photoperiod).

### Morphological Analysis

The entire developmental pathway and the embryonic stages were evaluated using a stereoscope (Leica, S8AP0), and micrographs were obtained using an inverted microscope (Leica, DMI4000). Samples were double-stained with acetocarmine and Evan’s blue (Gupta and Holmstrom, 2005). The dense cytoplasmic cells were stained into red, while vacuolated cells were stained into blue.

### Karyotype Analysis

Proembryogenic masses and more than 30 actively growing roots (1.5–2 cm long) of regenerated somatic-embryo derived seedlings were excised. The materials were pretreated in ice-cold water for 18–24 h and then fixed in Carnoy fixative (95% ethanol:acetic acid, 3:1) for 24 h, and then dissociated in 45% (v/v) acetic acid for 2 h. Finally, carbol-fuchsin staining was used for squash preparations, and then slides were observed and photographed using the cell workstation software Leica 4000. The chromosome number and type were determined for >25 cells from squashed Chinese fir root tips. Photos showing well-spread chromosomes were processed further using Photoshop CS3 and CAD 2010. The chromosome length in five cells was averaged.

### Microsatellite Analysis: Preparation of Genomic DNA and PCR Amplification

More than 30 regenerated plants of *C. lanceolata* and three proliferating PEMs were sampled for DNA isolation. Each sample was immediately frozen in liquid nitrogen and stored at -80°C until the DNA extraction. Samples were ground to a powder in liquid nitrogen, and genomic DNA was extracted using the DNeasy Plant Mini Kit (Qiagen).

We randomly selected 10 primer pairs from available nuclear microsatellite primers that had already been tested in *C. lanceolata* to detect genetic stability (see Supplementary Table S1). These nuclear simple sequence repeat (SSR) microsatellite loci were CFeSSR23, CFeSSR35, CFeSSR63, CFeSSR72, CFeSSR98, CFeSSR234, CFeSSR278, CFeSSR284, CFeSSR312, CFeSSR352, and CFeSSR418. These primers were developed from the transcriptomic analysis of genotype 6421 (Xu et al., 2016).

PCR amplification was performed by adding ∼2 μL diluted genomic DNA to a cocktail, with a final volume of 10 μL containing 1× PCR buffer, 25 nmol MgCl_2_, 2 nmol dNTPs, 1 U Taq DNA polymerase (Takara), and 1 nmol forward and reverse primers. PCR reactions were performed on a Veriti 96-well Thermal Cycler (Applied Biosystems) using the following procedure: 4 min at 94°C, followed by 20 cycles of 45 s at 94°C, 45 s at the annealing Tm, and 60 s at 72°C, with a final 10 min extension at 72°C. PCR products were run on 8% (w/v) native polyacrylamide gels.

### Analysis of Endogenous Hormone Levels

Fresh seeds at different developmental stages of the genotypes 4098, 5009, 4009, Y21, and 27 were collected for the detection of endogenous hormones. Megagametophytes (1.0–2.0 g) that contained immature zygotic embryos were immediately frozen in liquid nitrogen and stored at -80°C until analysis.

The methods for extraction and purification of ABA, IAA, GA_3_, and zeatin riboside (ZR) were modified from those described by Bollmark et al. (1988) and He (1993). ELISA hormonal quantification was following the protocols described by Yang et al. (2001) and Teng et al. (2006).

### Quantitative Real-Time PCR (qRT-PCR)

To isolate the potential *SERK* and *WOX* genes, the transcriptome database of *C. lanceolata* produced by Huang et al. (2012), Qiu et al. (2013), and Wang et al. (2013) was screened using the NCBI tblastn algorithm using the SERK homologs in Arabidopsis, *Medicago*, and rice (see Supplementary Table S2), and the WOX homologs in *P. abies* (Palovaara et al., 2010; Hedman et al., 2013) and Arabidopsis (Hecht et al., 2001) (see Supplementary Table S3). The potential genes were named on the basis of their alignment to Arabidopsis (see Supplementary Table S4). The unrooted neighbor-joining phylogenetic trees were generated from the deduced protein sequences using MEGA5.2 with the help of the Jones-Taylor-Thornton (JTT) model in combination with the gamma-distributed rate model (JTT+G; gamma = 0.97). Bootstrap values from 1,000 replicates were indicated at each node.

Tissues at different developmental stages were collected. Total RNA was extracted and purified following the methods previously described by Lin et al. (2009). RNA (1 μg) was reverse-transcribed with oligo (dT) and random hexamer primers using a reverse transcription system (Promega). Verification was performed using qRT-PCR.

qRT-PCR was carried out using the LightCycler 480 System (Roche Applied Science). Each reaction was performed in a 20-μL final volume containing 10 μL of 2× LightCycler 480 SYBR Green I Master, 0.5 μL of each homolog-specific primer pair (see Supplementary Table S5) at 100 nM, 5.0 μL of diluted cDNA template, and 4.0 μL of ddH_2_O. Each reaction was run in triplicate with the appropriate negative controls. Amplification was conducted under the following conditions: activation for 5 min at 95°C, followed by 40 cycles of 10 s at 95°C, 15 s at 60°C, and 15 s at 72°C. Fluorescence detection was performed after the extension step. The melting curve, with a temperature gradient from 60 to 95°C, was used to further investigate the specificity of each qRT-PCR reaction, and the presence of a single PCR product was also verified by 2% agarose gel electrophoresis. The eIF-3 housekeeping gene was selected as the endogenous reference gene for the relative PCR quantification (Wang et al., 2013).

## Results

### Generation of Somatic Embryos in Chinese Fir

**FIGURE 1:**
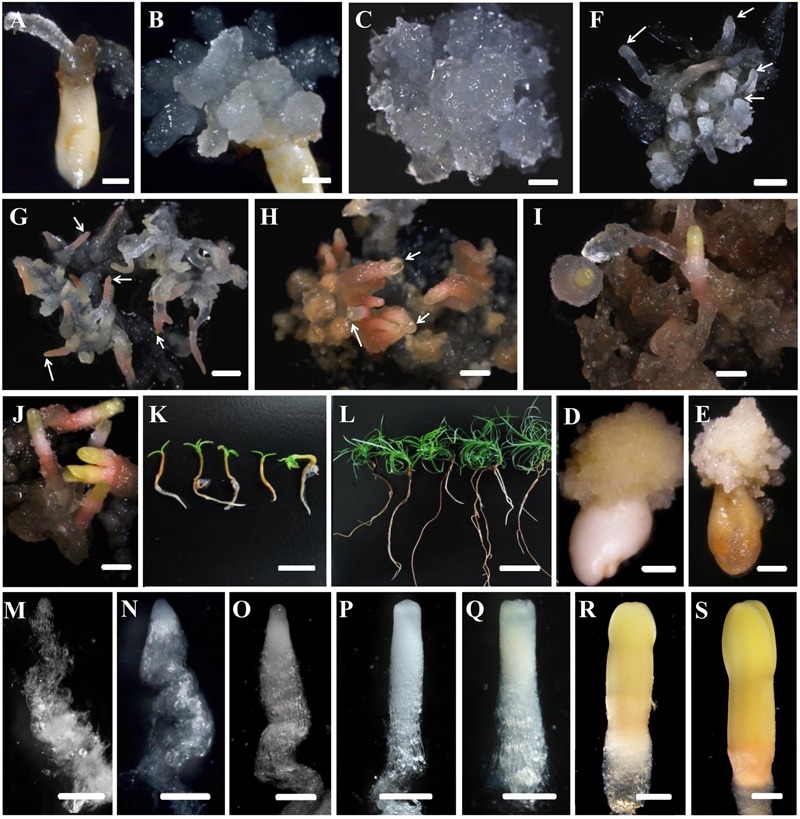
Developmental stages of SE **(A–L)** and zygotic embryogenesis **(M–S)** in *Cunninghamia lanceolata* (Lamb.) Hook. **(A,B,D,E)** Embryo sacs containing immature embryos for PEM induction: **(A,B)** induced embryogenic tissues; **(D,E)** induced non-embryogenic tissues. **(E,F–K)** Somatic embryo induction at diverse stages in Chinese fir: **(E)** maintenance of PEMs that were translucent and had pointed surface protrusions; **(F)** formation of proembryos (arrows); PEMs on a high osmolality medium for 1 week after suspension; end of proembryogeny; **(G)** shaped early reddish embryos (arrows); end of early embryogeny; **(H)** reddish embryos with bright yellow tops (arrows); the transition to late embryogeny; **(I)** early cotyledonary embryos; **(J)** late cotyledonary embryos; **(K,L)** seedlings germinated from somatic embryos at 1 week **(K)** and 1 month **(L)**. **(M–S)** Zygotic embryo development of Chinese fir based on Pullman and Webb (1994): **(M)** cleaved polyembryogeny before a dominant embryo forms; **(N)** proembryo, the beginning of early embryogeny; the dominant embryos has formed; **(O)** further developed dominant embryo with embryonal mass and suspensor prototypes; ready for late embryogeny; **(P)** dominant embryo with a more mature embryonal mass and suspensor; **(Q)** embryonal mass to cotyledon formation; **(R,S)** maturation of cotyledonary embryo. Bars = 1 mm for (**A–J)**; 500 μm for **(M–S)**; 1 cm for **(K,L)**.

**FIGURE 2:**
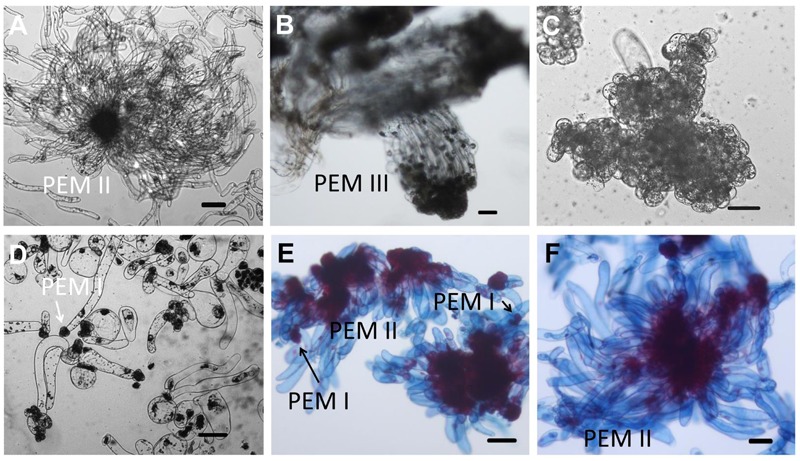
Microscopic structure of embryogenic and non-embryogenic tissues induced from immature embryos and suspension cultures in Chinese fir. Stage annotation based on Filonova et al. (2000). **(A)** Embryogenic tissues. Cell aggregates include a clump of rounded, dense cytoplasmic cells surrounded by a set of vacuolated cells that are elongated. **(B)** Well-organized PEM III structures after a more than half year maintenance period. **(C,D)** Non-embryogenic tissues also induced from immature embryos of Chinese fir: cell clusters composed of dense cytoplasmic cells but lacking elongated cells **(C)**, or with vacuolated cells in a collapsed cell shape but stuck in dense cytoplasmic cell aggregates (**D**, arrow represented PEM I structure). **(E,F)** Suspensions of PEMs stained with acetocarmine and Evan’s blue: **(E)** PEMs in suspension for 1 week, with more early-stage PEMs dedifferentiated (PEM I) (arrows); **(F)** PEMs in suspension for 3 weeks, with newly organized PEMs. Bars = 100 μm.

Our overarching objective was to develop an efficient system for Chinese fir mass propagation via SE. The appearance of PEMs with embryonic suspensor structures occurred on DCR medium containing a 2.0–6.0 mg L^-1^ 2,4-dichlorophenoxyacetic acid. Outgrowth occurred within 1–4 weeks when one or more zygotic embryos visibly protruded from the megagametophyte micropolar end into the medium (**Figure 1A**). One month later, outgrowths proliferated to form PEMs (**Figure 1B**). Embryogenic tissues were soggy and recognized as translucent structures, with pointed protrusions at the surface (**Figure 1C**). Microscopy of the cell aggregates revealed clumps of rounded, dense cytoplasmic cells surrounded by a set of vacuolated and elongated cells (**Figure 2A**), which were referred to as PEM II defined by Filonova et al. (2000). After a more than half year subculturing period, PEM II in the early stages became the assembled and well-organized PEM III (**Figure 2B**). However, non-embryogenic calluses looked quite different in color (**Figures 1D,E**), and cell clusters were not as well-organized (**Figures 2C,D**). Some non-embryogenic calluses showed a denser lump of cytoplasmic cells but lacked elongated cells (**Figure 2C**), whereas some contained vacuolated cells in collapsed shapes and were stuck in dense cytoplasmic cell aggregates (**Figure 2D**).

**FIGURE 3:**
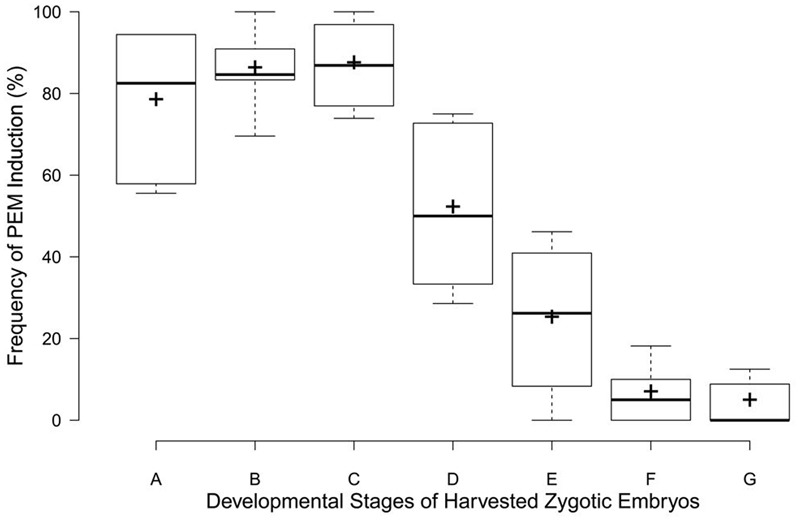
Box plots for PEM induction frequencies at different developmental zygotic embryo stages in *C. lanceolata*. More than 30 seeds were analyzed for each embryonic stage. Stage A, cleaved polyembryogeny stages (**Figure 1M**); stage B, proembryo, only a dominant embryo (**Figure 1M**); stage C, further developed dominant embryo (**Figure 1O**); stage D, dominant embryo had formed apical meristem primordium (**Figure 1P**); stage E, more changes occurred in embryonal mass to cotyledon formation (**Figure 1Q**); stages F and G, maturation of cotyledonary embryo (**Figures 1R,S**). Minimum and maximum PEM induction frequencies are depicted by short black lines, the box signifies the upper and lower quartiles, and the median and mean are respectively represented by a thicker black line and a thicker plus sign within the box for each stage.

The rate of PEM induction was related to the zygotic embryos’ developmental stage (**Figure 3**). The frequency was markedly higher for embryo sacs corresponding to early zygotic embryo stages (stages A–C, **Figure 3**), but sharply declined in late-stage embryos (stages D–E, **Figure 3**). Immature embryos in proembryogeny and early embryogeny were the best sources of explants for PEM induction.

The development of somatic embryos in Chinese fir is shown in **Figures 1F–L**. The maintained PEMs were transferred to liquid culture to synchronize the development of the cell masses and to produce vigorously differentiated late stage PEM structures. With the addition of ABA, PEMs separated into small groups of cell masses during the first week. At this point, early-stage PEMs, with fewer elongated cells, were seen, probably due to the shearing in the suspension (**Figure 2E**). Three weeks later, cell aggregates propagated both cell types and assembled into new PEM II structures (**Figure 2F**). Suspended PEMs were then dispersed into a high-osmolality environment to complete the critical transition from PEMs to proembryo, and to facilitate late embryogeny. Proembryos formed, having a dense embryonic head and highly vacuolated long polarized cells, but were still translucent (**Figure 1F**). In the following days, proembryos then turned red (**Figure 1G**), developed a bright yellowish top, and also increased in size (**Figure 1H**). A well-developed shoot and root pole took shape during this stage. Early cotyledonary-shaped embryos then formed, with the appearance of an indentation separating the developing cotyledon structures (**Figure 1I**). Finally, the two cotyledons fully opened, completing maturation (**Figure 1J**). Mature cotyledon embryos were generated on hormone and osmoticum (PEG) free medium (**Figures 1K,L**).

Investigation of the developmental processes of zygotic embryos indicated that somatic embryos went through analogous developmental phases. Suspension-cultured PEMs were synchronized and produced active PEM II cell aggregates (**Figure 2F**), which was the equivalent to cleaved zygotic embryo development (**Figure 1M**). During subsequent maturation, the zygotic embryo proper would then become white and translucent (**Figure 1N**). Both embryo and suspensor enlarged longitudinally (**Figure 1O**). Subsequently, the apical meristem primordium became visible (**Figure 1P**), followed by the appearance of cotyledon primordium (**Figure 1Q**). However, SE proembryos turned reddish rather than white (**Figure 1G**). When the SE embryo proper became opaque, the apical meristem primordium was clearly visible (**Figure 1H**). The cotyledon primordia formed accordingly (**Figure 1I**), but were not as easily distinguishable as in zygotic embryos (**Figure 1P**). On the whole, somatic embryos were smaller than zygotic embryos after they reached the proembryo stages.

### PEM Induction Frequency Correlated with Endogenous Auxin Level

**FIGURE 4:**
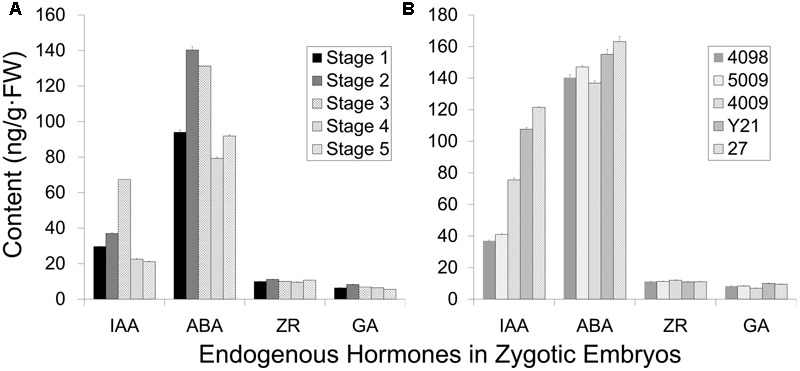
Endogenous hormone content of zygotic embryos (genotype 4098) at different developmental stages **(A)** and of different genotypes at the cleaved-embryo stage **(B)**. **(A)** The different developmental stages represented from 1 to 5: stage 1 cleaved polyembryos (**Figure 1M**); stage 2 proembryo (**Figure 1N**); stage 3 dominant embryo (**Figure 1O**); stage 4 columnar embryo (**Figure 1P**); stage 5 cotyledonary embryo (**Figures 1R,S**). **(B)** Genotypes 4098, 5009, 4009, Y21, and 27 were tested, and genotypes 4098 and 5009 were more competent to form somatic embryos. Data are means ± SD of three replicates.

The induction frequency of the PEMs was related to the developmental stages of the explants, as well as the plant genotypes. We investigated whether these differences correlated with endogenous hormonal levels using ELISA quantification. IAA and ABA levels oscillated during somatic embryo development, whereas ZR and GA_3_ levels were low and steady (**Figure 4A**). Cleaved embryos had the lowest IAA content during proembryogeny of zygotic embryo development (stage 1, **Figure 4**). The IAA concentration rose during development, until the dominant embryo formed opaque suspensors at the start of late embryogeny (stages 2–3, **Figure 4A**), and then a low level was maintained during late embryogeny until cotyledons emerged (stages 4–5, **Figure 4A**). The endogenous ABA content increased with the development of a dominant embryo and then declined during embryo maturation.

Endogenous IAA levels significantly varied among the genotypes of cleaved embryos (**Figure 4B**). Notably, genotypes 4098 and 5009 displayed relatively low levels of IAA at the stage of embryo cleavage, whereas IAA levels were higher in genotypes 4009, Y21, and 27 (**Figure 4B**). Not so significant differences in the ABA content were observed among the different genotypes. Genotypes 4098 and 5009 were effective sources for SE using our method, whereas the genotypes Y21, 4009, and 27 did not yield somatic embryos. Therefore, it appears that SE using this method may benefit from low endogenous IAA levels at the proembryogeny stage.

### ABA and PEG Are Different Stress Signals for Somatic Embryo Development

**FIGURE 5:**
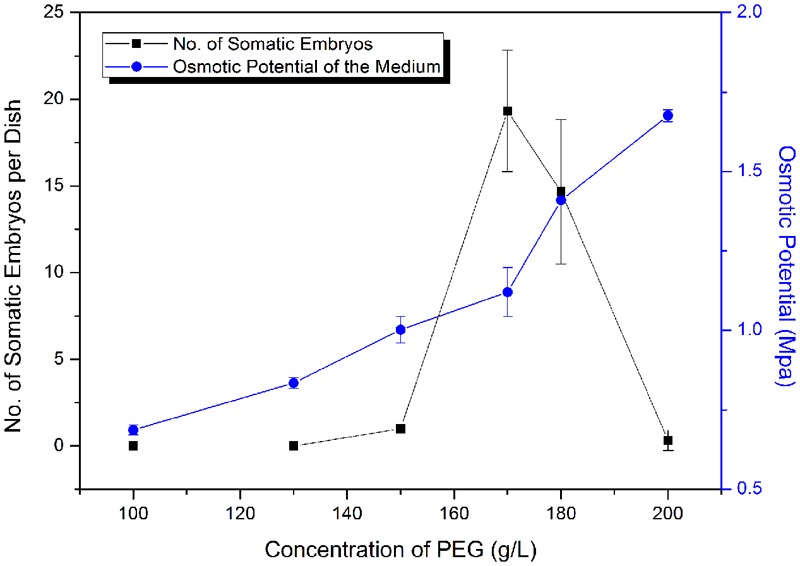
Frequency of somatic embryogenesis (SE) at different osmotic potentials. Data are means ± SD of three replicates.

Polyethylene glycol (MW 8000) is an important facilitator of somatic embryo maturation in conifer (Stasolla et al., 2003), which can mimic the desiccation that occurs during zygotic embryo maturation. Different concentrations of PEG (12–20%) were tested for effects on somatic embryo maturation (**Figure 5**). The number of somatic embryos increased sharply when PEG concentration increased from 150 to 170 g L^-1^ (15–17%), followed by a sharp decline at higher PEG concentrations (**Figure 5**). Thus, the optimized PEG concentration was 17%.

**FIGURE 6:**
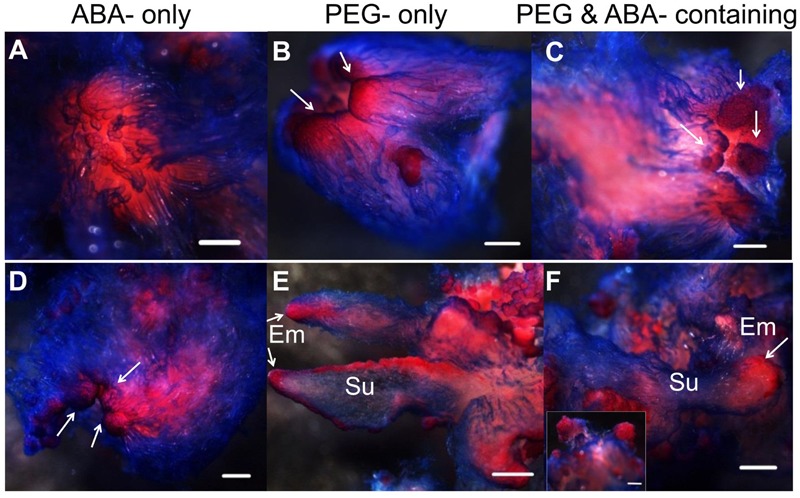
Influence of ABA and PEG on the formation of proembryos. Tissues were stained with acetocarmine and Evan’s blue and photographed under a stereoscope. **(A–C)** Incubated on maturation medium for 7 days; **(D–F)** incubated on maturation medium for 1 month; **(A,D)** incubated on ABA-containing medium; **(B,E)** incubated on PEG-containing medium; **(C,F)** incubated on ABA and PEG-containing medium. **(A)** No obvious changes occurred after 7 days incubation on ABA-containing medium; PEM II structure; **(B,C)** PEM III structures formed (arrows); **(D)** PEM III structures without clear bunchy-shaped vacuolated cells (arrows); **(E)** proembryos shaped with more dense cytoplasmic cells (arrows); **(F)** normal proembryos [insert, Em of proembryo (arrow)]. Su, suspensor; Em, embryonal mass. Bars = 500 μm.

Abscisic acid, “stress hormone” in plants, is also widely used during SE induction (Zavattieri et al., 2010). To further clearly elucidate the effect of high osmolality and exogenous ABA during SE, we compared the effects of PEG-, ABA-, and PEG and ABA- containing maturation media on morphological changes during embryo development (**Figure 6**). Differences in morphology between embryos on PEG-free (**Figure 6A**) and PEG-containing (**Figures 6B,C**) media were visible after the first 7 days of incubation. Polar PEM III structures were evident when PEG was present, whereas embryos on the ABA-only medium did not differentiate beyond PEM II. After 1 month, variations were more obvious. By that time, PEM III structures with polarity had formed in the ABA-only media, without clusters of vacuolated cells associated with proembryo formation (**Figure 6D**). PEG alone produced more proembryo-like structures; however, the suspensor consisted of not only vacuolated cells but also dense cytoplasmic cells (**Figure 6E**). Normal proembryos with embryonal masses from dense cytoplasmic cells and suspensors from vacuolated cells were obtained only on medium containing both PEG and ABA (**Figure 6F**). In total, we found that ABA contributed the most to embryonal mass development, whereas PEG facilitated suspensor development and orientation.

### Karyotype and Microsatellite Analyses Reveal True-to-Type Propagation via SE

A karyotype analysis of plants regenerated through SE was performed by assessing >30 actively growing roots from 30 different individuals. All of the regenerated plants were diploids with 22 chromosomes (2n = 2X = 22; see Supplementary Figure S1A). The relative length and the ratio of the long arm to the short arm (see Supplementary Figure S1B and Supplementary Table S6) indicated that the karyotype belonged to 1B and consisted of 10 pairs of metacentric and 1 pair of submetacentric chromosomes.

Eleven randomly selected primers developed from a transcriptome analysis of genotype 6421 were used to explore the microsatellite stability of the regenerated plants (see Supplementary Table S1). All primers exhibited monomorphic bands between the 3 tested PEM lines and 30 regenerated plants (see Supplementary Figure S2).

### SERK and WOX May Serve As Markers of Stress-Mediated Cell-Signaling during SE

Somatic embryogenesis is also recognized as a reprogrammed developmental process responding to stress-related signals (Zavattieri et al., 2010). Several gene regulators of this process have been identified. SERK is considered to be the critical switch from somatic to embryogenic development, and WUS maintains a small group of cells at the dedifferentiation state (Smertenko and Bozhkov, 2014). Thus, we examined the expression patterns of these two widely recognized gene markers of early embryogenesis during SE.

As there was little gene information for Chinese fir, sequences of SERK and WOX from *P. abies*, Arabidopsis, maize, rice, and alfalfa were selected for screening against the transcriptome of Chinese fir 6421. We obtained five orthologous contigs each for SERK and WOX (see Supplementary Table S4). Neighbor-joining phylogenetic analysis and qRT-PCR were conducted on both genes.

**FIGURE 7:**
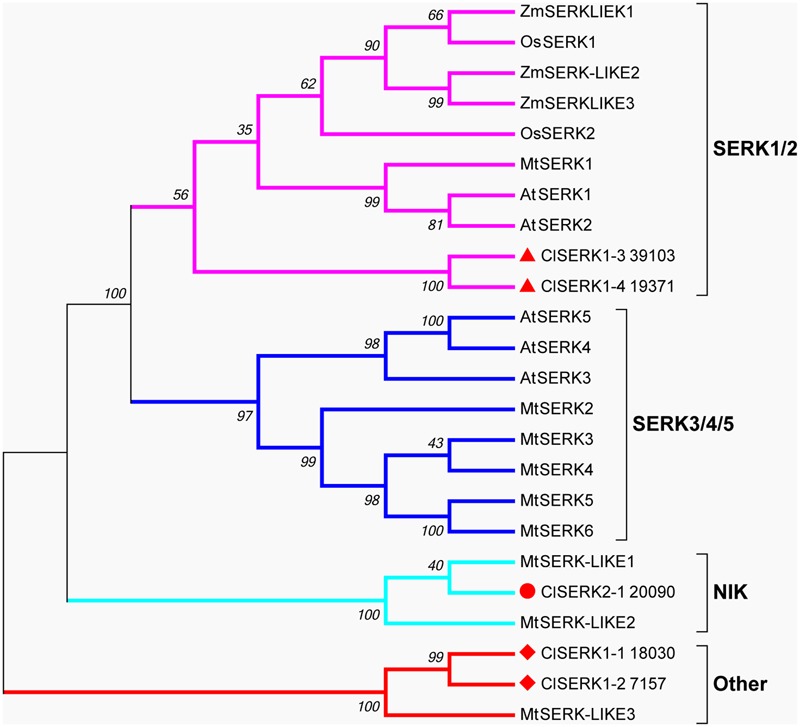
Neighbor-joining phylogenetic tree of full-length SERK and SERK-like protein sequences generated using the Jones-Taylor-Thornton (JTT) model and gamma-distributed (gamma = 0.97) with 1,000 bootstrap replicates. Four branches, SERK1/2, SERK3/4/5, NIK and Other are marked with different colors. At: *Arabidopsis thaliana*; Cl: *Cunninghamia lanceolata*; Mt: *Medicago truncatula*; Os: *Oryza sativa*; Zm: *Zea mays*. Accession numbers are provided in Supplementary Table S2.

**FIGURE 8:**
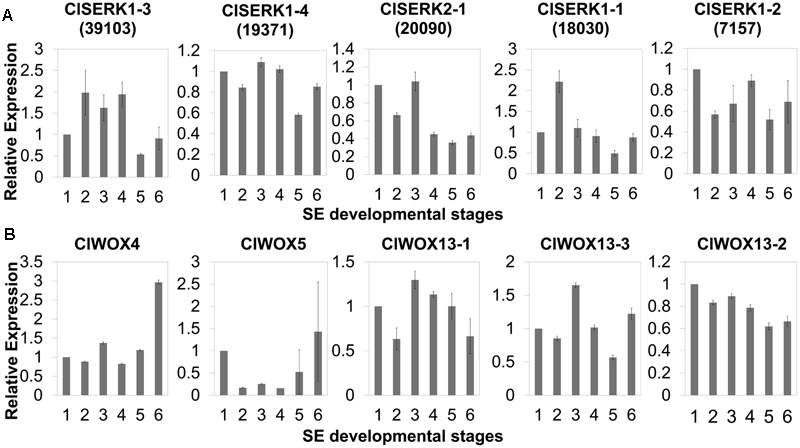
qRT-PCR expression studies of putative *SERK* **(A)** and *WOX* **(B)** genes during the six developmental stages of SE in Chinese fir. The six developmental stages are: (1) PEMs maintained for 20 days, as seen in **Figure 1C**; (2) PEMs suspended for 2 days; (3) PEMs suspended for 3 weeks, as seen in **Figure 2F**; (4) proembryos grown on maturation medium for 1 week, as seen in **Figure 1F**; (5) reddish proembryos, as seen in **Figure 1G** or 1H; (6) cotyledon embryos, as seen in **Figure 1J**. Results shown are means ± standard errors of three replicates, calibrated to the expression in the starting PEMs (1).

The phylogenetic tree of SERK showed four main branches (**Figure 7**), annotated as SERK1/2, SERK 3/4/5, nuclear shuttle protein interacting kinase (NIK), and Other (Nolan et al., 2011). The SERK1/2 group included two ClSERK1 (39103 and 19371) contigs. MtSERKL1, MtSERKL2, and ClSERK2-1 (20090) were in the subclade of the third group and were more similar to NIK in Arabidopsis. The fourth subgroup included ClSERK1-1 (18030), ClSERK1-2 (7157), and MtSERKL3. In general, the relative expression data showed that the ClSERKs transcript levels were the highest during the development of the proembryos and the lowest in developed embryos (**Figure 8A**). *ClSERK1-3* (39103) transcript levels were almost twofold higher at the end of proembryogeny (stage 4) compared with embryogenic calluses (stage 1), followed by a sharp decrease during late embryogeny (stages 5 and 6). *ClSERK1-4* (19371), although evolutionarily close to *ClSERK1-3* (39103), had a different expression pattern. *ClSERK1-1* (18030) transcript levels rose significantly when PEMs were embedded in the suspension containing ABA for differentiation (stage 2), but then declined, reaching an even lower level in PEMs when the dominant embryos began to develop (stage 5). *ClSERK2-1* (20090) and *ClSERK1-2* (7517) exhibited higher expression levels in the PEMs (stage 1) than in the developed embryos (stages 4, 5, and 6).

**FIGURE 9:**
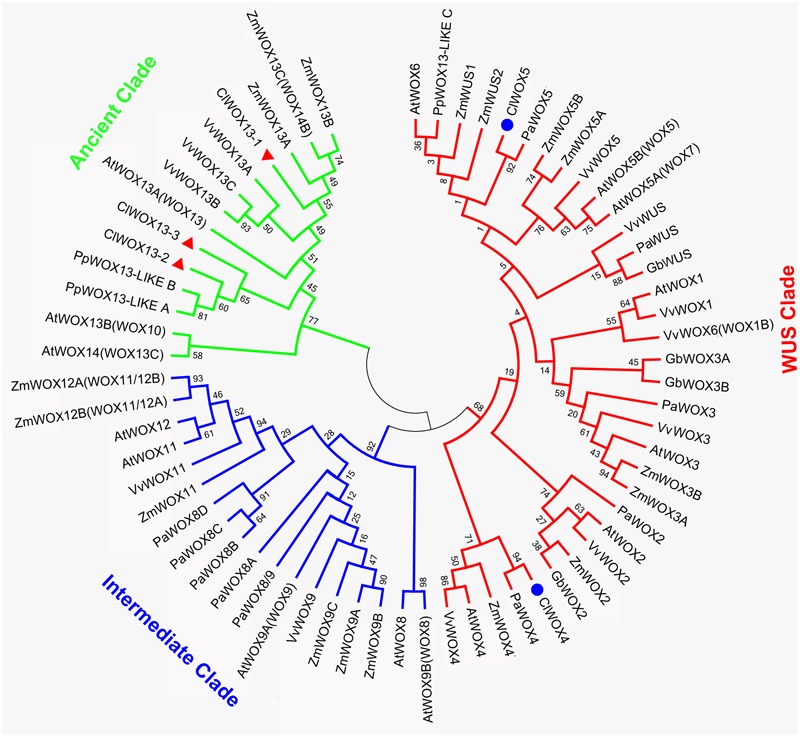
A phylogenetic analysis of full-length WOX protein sequences was performed using the neighbor-joining statistical method and the Jones-Taylor-Thornton (JTT) model, and was gamma-distributed (gamma = 0.97) with 1,000 bootstrap replicates. At: *Arabidopsis thaliana*; Cl: *Cunninghamia lanceolata*; Gb: *Ginkgo biloba*; Mt: *Medicago truncatula*; Os: *Oryza sativa*; Pa: *Picea abies*; Pp: *Physcomitrella patens*; Vv: *Vitis vinifera*; Zm: *Zea mays*. Accession numbers are provided in Supplementary Table S3.

The unrooted tree of the plant WOX proteins (**Figure 9**) can be naturally divided into three clades, referred to as the WUS, intermediate, and ancient clades (van der Graaff et al., 2009). The ClWOX4 group contains PaWOX4, and the ClWOX5 group contains PaWOX5. Both are divergent from the angiosperm WOX genes. Three different ClWOX13 contigs were included in the WOX13 clade, the most ancient WOX clade (van der Graaff et al., 2009). ClWOX13-2 and ClWOX13-3 were closely related to two WOX13-like proteins of the non-vascular moss *Physcomitrella patens*, whereas ClWOX13-1 was closer to the three maize WOX13s. qRT-PCR results (**Figure 8B**) revealed that *ClWOX13-1* and *ClWOX13-3* transcript levels were gradually up-regulated in liquid suspensions containing ABA (from stages 2 to 3), and decreased during proembryogeny (stage 4) to early embryogeny (stage 5). The expression of *ClWOX4* and *ClWOX5* was comparatively high during late embryogeny (stage 6), which may be due to their functions in the procambia of cotyledon embryo and root stem cells, respectively. *ClWOX13-2* transcript level was lowest at the end of early embryogeny (stage 5) and had a smaller peak during embryo maturation (stage 6).

## Discussion

### An Efficient Synchronized SE System of Chinese Fir Was Developed to Regenerate True-to-Type Somatic-Embryo Derived Plants

Somatic embryogenesis of *C. lanceolata* has the potential to produce a large-scale supply of excellent seedlings for plantation development in commercial timber resources strategic reserve, and it is an attractive model for studying early embryogenic events due to the inability to access to zygotic embryos. Tissue culture of Chinese fir via adventitious buds multiplication began in the late 1970s (Que, 1980) and continues to advance (Wang, 1999; Zhu et al., 2007a; Zhou et al., 2013). Recently, Li et al. (2017) reported on the production of plantlets from leaf cuttings. While, development of an SE system for *C. lanceolata* has also been ongoing for years, much less has been reported on that progress. Three method improvements have recently been published for Chinese fir SE. (1) Explants of cotyledon, hypocotyl and mature embryos (Xi and Shi, 2005, 2006) were used to directly induced somatic embryos, but with a low propagation rate of <10%, which is not suitable for mass propagation; (2) Hu et al. (2017) induced embryos indirectly from calluses initiated from immature dominant zygotic embryos by successive culturing on a low auxin/cytokinin concentration medium. This methodology can produce embryos at much higher rate, but somatic embryos may be contaminated with adventitious buds via indirect organogenesis that are not readily distinguished. (3) Hu et al. (2017) also used ABA and PEG to induce somatic embryos starting with embryogenic calluses from immature cleavage polyembryony-staged embryos. Although they obtained mature embryos, these embryos were recalcitrant to obtain normal cotyledon embryos and regenerate plantlets. In our studies, the yield and improved uniformity of somatic embryos offer a more stable SE system for mass propagation in Chinese fir. Moreover, we synchronized PEM development by introducing a liquid suspension system before maturation to proembryos was initiated (Chen and Chen, 2007). From the standpoint of operational economics, this system is cost-efficient, significantly shortening the propagation circulation time from 3 to 1 week. What’s more, the proembryos developed quite analogously to zygotic embryos, and offer great promise for conifer embryo development and transgenic system studies.

For long-living forest trees, somaclonal variation can significantly affect the quality of cloned individuals, and can result in severe economic losses years after planting. It frequently occurs due to *in vitro* culture conditions, such as high concentrations of growth regulators and long-term culturing (DeVerno et al., 1999). In our study, high hormone concentrations during PEM maintenance and proembryo induction, high osmolality induced by PEG, and a relatively long maturation term may contribute to the higher risk of somaclonal variation. Ploidy level of the regenerants via SE was therefore determined by karyotype analysis. In addition all SSR primers used showed monomorphic bands, suggesting our “true-to-type” propagation system.

### Endogenous Hormonal Changes Revealed Correlation between Seed Developmental Stages, Genotypes, and Competence Acquisition for SE

The induction of PEMs is critical during the whole SE process as it may determine the PEM production efficiency and the capability of SE, which probably relies on the seed’s developmental stage and genotypic selection. The strategic selection of competent genotypes and explants during the collection period for PEM induction could significantly reduce production cost and time. In conifers, somatic embryogenic tissues are always induced from juvenile tissues. For most pine species, early-stage immature embryos embedded in megagametophytes are highly competent for SE (Stasolla and Yeung, 2003; Salajova and Salaj, 2005; Park et al., 2006). In *C. lanceolata*, a high efficiency of PEM induction was obtained using proembryo-stage embryos (**Figure 3**). However, only two PEM lines induced from cleaved embryos ultimately produced mature somatic embryos, whereas PEMs of other older stages failed using the same protocol. A large body of experimental observations exists on the central role of endogenous IAA in the regulation of embryo development (Jenik and Barton, 2005). Auxinic herbicide (2,4-D) is widely applied for embryogenic tissue induction, but removal of 2,4-D is critical at certain stages of cultured embryo development. In Arabidopsis culture, removal of 2,4-D induces the expression of *YUCCA* genes. *YUCCA*s encode key enzymes in auxin biosynthesis which increase endogenous IAA levels (Bai et al., 2013), which in turn can trigger IAA polar transport (Su et al., 2009). The addition of an auxin antagonist PCIB (*p-*chlorophenoxy isobutyric acid) to maturation medium prevents embryogenic tissue proliferation and prompts the development of mature embryos. Moreover, the addition of the auxin synergist phloroglucinol increases the proliferation of embryogenic tissues and totally suppresses the maturation. This may indicate that endogenous IAA at high levels can improve proliferation but block maturation of embryogenic cultures of Nordmann fir (Find et al., 2002). The PCIB treatment was also conducted in Scots pine, which reduced the proliferation of embryogenic tissues and significantly increased the yield of cotyledon embryos (Abrahamsson et al., 2011; Park and Bonga, 2013). There is more detailed understanding of auxin responses in somatic embryo formation in Norway spruce. Addition of the polar auxin transport inhibitor 1-*N*-naphtylphthalamic acid (NPA) (Benková et al., 2003) caused endogenous IAA content to increase in early embryos, but programmed cell death (PCD) decreased and differentiation of the suspensor was abnormal (Larsson et al., 2008). During late embryo formation, NPA-treatment resulted in aberrant apical and basal patterning in mature embryos (Larsson et al., 2008; Palovaara et al., 2010). Therefore, maintenance of auxin transport and avoidance of local auxin buildups is important for the transition of embryogenic tissues to proembryos. In our study, we found that cleaved embryos contained the lowest levels of IAA prior to the emergence of the dominant embryos (**Figure 4A**). Moreover, genotypes 4098 and 5009, with higher competence levels for SE, exhibited lower cleaved embryo stage IAA levels than the recalcitrant genotypes Y21, 4009, and 27 (**Figure 4B**). The same trend between SE competence and genotype was also discovered in other conifer. Pre-and post-cleavage stages before the appearance of a dominant embryo also showed the strongest responses to somatic embryo initiation in *P. strobus* (Park, 2002). In *Picea morrisonicola* Hayata, a less competent line had a 7.5-fold higher endogenous IAA content than a competent line, and a reduction in the endogenous IAA content during proliferation improved the somatic embryo induction competence for the poorly inducing line (Liao et al., 2008). We hypothesize that lower endogenous auxin levels at early somatic embryo developmental stages can facilitate the acquisition of competence during SE. It is widely known that the potential of SE is genotype dependent. Many factors influencing SE competence acquirement has been investigated during SE development, such as antioxidants, amino acids, calcium irons, salicylic acid, genetic cues, and so on (Teixeira da Silva and Malabadi, 2012). We revealed high correlation between endogenous IAA level in juvenile explants and genotype competence. Screening the endogenous IAA level in the initial explants had applicability as a useful tool for identifying genotypes that are appropriate for SE. However, more experiments need to be conducted for further verification.

### ABA and PEG Play Complementary Role in Proembryo Formation

Osmotic changes are critical during SE competence acquisition. In most coniferous species, exogenous ABA is necessary to stop cell proliferation and stimulate somatic embryo development (Zavattieri et al., 2010; Jain et al., 2013). ABA induces cells’ desiccation tolerance from a hormonal level (Bozhkov et al., 2002), whereas PEG causes osmotic changes that affect cells turgor from physiological aspect (Kermode, 1990). Most reports focused on the quality improvement by ABA especially for promoting late embryogeny (Bozhkov and Von Arnold, 1998; Bozhkov et al., 2002; Aronen et al., 2009). In our case, exogenous ABA triggered PEM differentiation and promoted sustained vigor in suspension cultures. Moreover, morphological effects of PEG and ABA on embryogenesis were compared. We conducted such an investigation using ABA-, PEG-, and ABA and PEG-containing maturation media. The results demonstrated that PEG initiated morphological changes in the first week, promoting polarity in PEM III (**Figures 6B,C**). After a 1-month incubation, normal proembryos were obtained only on the medium containing ABA and PEG (**Figure 6F**). The ABA-only medium produced enlarged embryonal masses without clusters suspensors (**Figure 6D**), whereas proembryo-like structures lacking enlarged embryonal masses were produced under PEG-only condition (**Figure 6E**). Thus, ABA and PEG contribute in distinct and perhaps in interacting ways to proembryo formation. In addition, the early addition of ABA could delay the development of PEM III, which explains why newly formed somatic embryos in Norway spruce grow well on plant growth regulator–free medium for at least 1 week (von Arnold et al., 2005).

### Molecular Evidence Uncovered Early Stress-Related Signaling during SE Development

From PEMs to somatic embryos, each transition stage involves physiological and metabolic alterations. These changes were powered by gene regulators. *SERK* and *WUS* are two widely recognized gene markers of early embryogenesis. Recent progress in cotton has confirmed the role of SERK in cellular redox related somatic cell-to-embryo transition (Pandey and Chaudhary, 2014). WUS are responsible for keeping cell undifferentiated state (Laux et al., 1996).

*SERK* gene family attributes to the leucine-rich repeat (LRR) subfamily that coded for a transmembrane protein involved in signal transduction that was strongly related to stress responses of SE (Santos and Aragão, 2009). AtSERK1 from Arabidopsis had direct function on SE and determined the embryogenic competence in culture (Hecht et al., 2001). In *Medicago, MtSERK1*, orthologous to *AtSERK1*, was up-regulated by auxin and functioned broader than embryogenesis alone (Nolan et al., 2003). In monocots, *OsSERK1* can response to defense signaling hormonal activity, such as salicylic acid, jasmonic acid, and ABA (Hu et al., 2005). Phylogenetic results showed that two ClSERK1 contigs (39103 and 19371), which are predominantly expressed in PEMs, shared a high similarity with AtSERK1 and MtSERK1, indicating that ClSERK1-3 and ClSERK1-4 may function uniformly in SE. MtSERKL1 and MtSERKL2 are more similar to NIK genes in Arabidopsis (Nolan et al., 2011). Arabidopsis NIK shares many similarities with SERK; and is the transducer of a novel layer of plant innate defenses (Santos et al., 2010). ClSERK2-1 (20090), grouped together with MtSERKL1 and MtSERKL2, had higher transcript levels in stages 1 and 3 (**Figure 8**), based on which, we postulated that the transition to late stage PEM may come with their stress related responses.

*WUS* is the prototypical member of the *WOX* family. *WUS* overexpression increases the acquisition of embryogenic competence in Arabidopsis (Zheng et al., 2014) and cotton (Zuo et al., 2002). In Arabidopsis zygotic embryo development, WOX2, 8, and 9 regulate cell fate by determining the early apical and basal patterning events (Haecker et al., 2004). In conifer, *PaWOX2, WOX8/9, PIN-FORMED* (*PIN*) and *polar auxin transport* (*PAT*) regulate embryo patterning during embryo development (Palovaara et al., 2010). Expression of WOX2, 8, and 9 orthologs was not apparently linked to Chinese fir embryo development, but three ClWOX13 contigs that grouped into the ancient WOX13 clade (**Figure 9**). *ClWOX13* exhibited significantly higher transcript levels at stage 3, concurrent with the proembryo transition (**Figure 8B**). ClWOX13-1 was more closely related to the three maize WOX13, whereas the other two, ClWOX13-2 and ClWOX13-3, clustered with two WOX13-like genes of the non-vascular moss *P. patens* (**Figure 9**). Interestingly, recent studies in *P. patens* elucidated the function of two *PpWOX13*-like genes in the control of cell wall loosening to facilitate stem cell formation, but no regeneration-related functions has yet been reported for the *AtWOX13* (Sakakibara et al., 2014). qRT-PCR results revealed that expression of *ClWOX13* may have high correlation with stage 3, which is when the critical transition to proembryo occurs. Because new evidence indicates that *Cunninghamia* is a long and widespread living fossil that extends back to the mid-Mesozoic period (Shi et al., 2014), we postulate that *ClWOX13* may still conserve some functions of embryogenesis regulation in Chinese fir. Early *WOX* transcriptional machinery correlates with the gradient endogenous auxin distribution through the expression of the auxin transport-related genes (Breuninger et al., 2008), and *SERK* also is auxin-induced (Nolan et al., 2003). From this aspect, it will be of particular interest to explore the connection between the *WOX* and *SERK* functions and endogenous auxin during early embryogenesis in Chinese fir.

## Conclusion

In summary, our study provides an efficient synchronized SE system via a liquid suspension in Chinese fir that also might lead to the improvement of SE in other gymnosperms. Karyotyping and microsatellite analysis support preliminary optimism that no major cytological or genetic variation occurred in our plants regenerated via SE. A hormonal analysis of IAA, ABA, ZR, and GA_3_ uncovered a potential relationship between endogenous IAA and genotypic recalcitrance. Lower endogenous IAA of early stage immature embryos tends to higher competence acquisition. Morphological studies of PEMs during maturation revealed that ABA and PEG play complementary roles during proembryo formation. ABA boosted the growth of embryonal masses, whereas PEG functioned on the organization of proembryo-like structures. Finally, the phylogenetic tree and expression patterns suggested the probable regulation and activities of *SERK* and *WOX* during SE, especially in early stages of proembryo formation. SERK is conserved and redundantly expressed in SE, whereas WOX13 is the most promising candidate for exploring the function of WOX in Chinese fir embryo development.

## Author Contributions

JC, JS, and TL conceived and designed the experiments, XZ performed experiments and analyzed data, RZ prepared all the samples, GL performed the cytological analysis, YX helped to pick up SSR primers and data analysis. YwZ contributed to plant materials’ subculture, YZ helped with RT-PCR data collection, XZ and RZ wrote the manuscript with contributions from all authors, TL and SH contributed to the manuscript revision.

## Conflict of Interest Statement

The authors declare that the research was conducted in the absence of any commercial or financial relationships that could be construed as a potential conflict of interest. The reviewer TP declared a shared affiliation, with no collaboration, with one of the authors, TL, to the handling Editor.
