# Supplementary material for: Desiccation Treatment and Endogenous IAA Levels Are Key Factors Influencing High Frequency Somatic Embryogenesis in Cunninghamia lanceolata (Lamb.) Hook
Source: Front Plant Sci. 2017 Dec 5;8:2054. doi: 10.3389/fpls.2017.02054 (PMC5723420; doi:10.3389/fpls.2017.02054)
Supplement: Supplementary file 1 [file Table_1.DOCX]

Supplementary Material

Desiccation treatment and endogenous IAA levels are key factors influencing high frequency somatic embryogenesis in *Cunninghamia lanceolata* (Lamb.) Hook

Xiaohong Zhou^1,2†^, Renhua Zheng^3†^, Guangxin Liu^1,2^, Yang Xu^1‡^, Yanwei Zhou^1,2^, Thomas Laux^4^, Yan Zhen^1,2^, Scott A. Harding^5^, Jisen Shi^1,2*^, and Jinhui Chen^1,2*^

*** Correspondence:** Dr. Jinhui Chen: Tel.: +86 25 85428817; E-mail: chenjh@njfu.edu.cn; Dr. Jisen Shi: Tel.: +86 25 85428948; Fax: +86 25 85428948; E-mail: jshi@njfu.edu.cn.

## Supplementary Tables

**Supplementary Table S1**. SSR primers used to assess the genetic stability of PEMs and plants regenerated via SE.

| **Locus^a^** | **T_m_** | **Allele size** | **Primer (5′–3′)** |
| --- | --- | --- | --- |
| CFeSSR23 | 50 | 101 | F:GCTATCATCGACACCAAC  R:CACTTCACCAAACTCTTCT |
| CFeSSR35 | 53 | 416 | F:TCCTTCCTTCCGTGTCTA  R:CAATGAATATGATGATGGGT |
| CFeSSR63 | 54 | 237 | F:CTTCCGACTTCACCAAAC  R:TCCAATAAGCATAGCCAC |
| CFeSSR72 | 49 | 297 | F:TCTCACGGTGCTCTGTTGG  R:TGTCAGGGCGATTGTTGC |
| CFeSSR98 | 54 | 361 | F:AAACTCCCTGTAGACCCA  R:GCTCTGCTAAGCGTATGTC |
| CFeSSR234 | 54 | 499 | F:CGTCATCCTGGGATCTTT  R:GTTAGTGGCTTGCTTACC |
| CFeSSR278 | 49 | 359 | F:TGGTTAGCCAGGAAAGCC  R:GGGAATGGTGGGAGGAAT |
| CFeSSR284 | 49 | 108 | F:GCAGGAACCACAGATACACTC  R:TTCGGCTACTGATAGGACTT |
| CFeSSR312 | 50 | 279 | F:CCTGCTGACACGCCTAAA  R:GGCCAAGAGTGCCTTCTA |
| CFeSSR352 | 50 | 329 | F:TTCCCTCAACCAAATCCTT  R:GATCCCTCCATTGCAGTTT |
| CFeSSR418 | 50 | 262 | F:CGAACCACGGTTATTTCT  R:CCAGGGACTTCTTCAGCA |

T_m_ , melting temperature

^a^SSR primers developed from transcriptome analysis of genotype 6421.
