# Supplementary material for: Desiccation Treatment and Endogenous IAA Levels Are Key Factors Influencing High Frequency Somatic Embryogenesis in Cunninghamia lanceolata (Lamb.) Hook
Source: Front Plant Sci. 2017 Dec 5;8:2054. doi: 10.3389/fpls.2017.02054 (PMC5723420; doi:10.3389/fpls.2017.02054)
Supplement: Supplementary file 2 [file Table_2.DOCX]

Supplementary Material

Desiccation treatment and endogenous IAA levels are key factors influencing high frequency somatic embryogenesis in *Cunninghamia lanceolata* (Lamb.) Hook

Xiaohong Zhou^1,2†^, Renhua Zheng^3†^, Guangxin Liu^1,2^, Yang Xu^1‡^, Yanwei Zhou^1,2^, Thomas Laux^4^, Yan Zhen^1,2^, Scott A. Harding^5^, Jisen Shi^1,2*^, and Jinhui Chen^1,2*^

*** Correspondence:** Dr. Jinhui Chen: Tel.: +86 25 85428817; E-mail: chenjh@njfu.edu.cn; Dr. Jisen Shi: Tel.: +86 25 85428948; Fax: +86 25 85428948; E-mail: jshi@njfu.edu.cn.

## Supplementary Tables

**Supplementary Table S2.** Accession numbers of the SERK protein sequences used for phylogenetic analysis. At: *Arabidopsis thaliana*, Mt: *Medicago truncatula*, Os: *Oryza sativa*, Zm: *Zea mays*. (Nolan et al., 2003; Hu et al., 2005; Singla et al., 2009; Nolan et al., 2011)

| **Gene name** | **Accession no.** | **Gene name** | **Accession no.** |
| --- | --- | --- | --- |
| At_SERK1 | AEE35238 | Mt_SERK1 | AAN64293 |
| At_SERK2 | AEE31686 | Mt_SERK2 | ADO15291 |
| At_SERK3 | AF384970 | Mt_SERK3 | ADO15298 |
| At_SERK4 | AEC06259 | Mt_SERK4 | ADO15292 |
| At_SERK5 | AEC06260 | Mt_SERK5 | ADO15293 |
| Zm_SERK-LIKE1 | CAC37640 | Mt_SERK6 | ADO15294 |
| Zm_SERK-LIKE2 | CAC37641 | Mt_SERK-LIKE1 | ADO15295 |
| Zm_SERK-LIKE3 | CAC37642 | Mt_SERK-LIKE2 | ADO15296 |
| Os_SERK1 | AAU88198 | Mt_SERK-LIKE3 | ADO15297 |
| Os_SERK2 | BAD86793 |  |  |

**Reference**

Hu, H., Xiong, L., and Yang, Y. (2005). Rice *SERK1* gene positively regulates somatic embryogenesis of cultured cell and host defense response against fungal infection. *Planta* 222(1)**,** 107-117.

Nolan, K.E., Irwanto, R.R., and Rose, R.J. (2003). Auxin up-regulates *MtSERK1* expression in both *Medicago truncatula* root-forming and embryogenic cultures. *Plant Physiology* 133(1)**,** 218-230.

Nolan, K.E., Kurdyukov, S., and Rose, R.J. (2011). Characterisation of the legume *SERK-NIK* gene superfamily including splice variants: implications for development and defence. *BMC Plant Biol* 11**,** 44.

Singla, B., Khurana, J.P., and Khurana, P. (2009). Structural characterization and expression analysis of the *SERK/SERL* gene family in rice (*Oryza sativa*). *Int J Plant Genomics* 2009**,** 539402.
