# Supplementary material for: Desiccation Treatment and Endogenous IAA Levels Are Key Factors Influencing High Frequency Somatic Embryogenesis in Cunninghamia lanceolata (Lamb.) Hook
Source: Front Plant Sci. 2017 Dec 5;8:2054. doi: 10.3389/fpls.2017.02054 (PMC5723420; doi:10.3389/fpls.2017.02054)
Supplement: Supplementary file 3 [file Table_3.DOCX]

Supplementary Material

Desiccation treatment and endogenous IAA levels are key factors influencing high frequency somatic embryogenesis in *Cunninghamia lanceolata* (Lamb.) Hook

Xiaohong Zhou^1,2†^, Renhua Zheng^3†^, Guangxin Liu^1,2^, Yang Xu^1‡^, Yanwei Zhou^1,2^, Thomas Laux^4^, Yan Zhen^1,2^, Scott A. Harding^5^, Jisen Shi^1,2*^, and Jinhui Chen^1,2*^

*** Correspondence:** Dr. Jinhui Chen: Tel.: +86 25 85428817; E-mail: chenjh@njfu.edu.cn; Dr. Jisen Shi: Tel.: +86 25 85428948; Fax: +86 25 85428948; E-mail: jshi@njfu.edu.cn.

## Supplementary Tables

**Supplementary Table S3.** Accession numbers and genome loci of the WOX sequences used for phylogenetic analysis. At: *Arabidopsis thaliana*, Mt: *Medicago truncatula*, Os: *Oryza sativa*, Pa: *Picea abies*, Pp: *Physcomitrella patens*, Vv: *Vitis vinifera*, Zm: *Zea mays*, Gb: Ginkgo biloba.(Haecker et al., 2004; Deveaux et al., 2008; Gambino et al., 2011; Hedman et al., 2013)

| **Gene name** | **Accession no./Locus** | **Gene name** | **Accession no.** |
| --- | --- | --- | --- |
| Pa_WUS | JX512364 | Zm_WUS1 | AM234744 |
| Pa_WOX2 | AM286747 | Zm_WUS2 | AM234745 |
| Pa_WOX3 | JX411947 | Zm_WOX2 | AM490235 |
| Pa_WOX4 | JX411948 | Zm_WOX3B | AM491777 |
| Pa_WOX5 | JX411949 | Zm_WOX3A | AM490236 |
| Pa_WOX8/9 | GU944670 | Zm_WOX4 | AM490237 |
| Pa_WOX8A | JX411950 | Zm_WOX5A | AM490238 |
| Pa_WOX8B | JX411951 | Zm_WOX5B | AM490239 |
| Pa_WOX8C | JX411952 | Zm_WOX9A | AM490240 |
| Pa_WOX8D | JX411953 | Zm_WOX9B | AM490241 |
| At_WOX1 | AT3G18010 | Zm_WOX9C | AM490242 |
| At_WOX2 | AT5G59340 | Zm_WOX11 | ACG26290 |
| At_WOX3 | AT2G28610 | Zm_WOX12A(ZmWOX11/12A) | AM234774 |
| At_WOX4 | AT1G46480 | Zm_WOX12B(ZmWOX11/12B) | AM234775 |
| At_WOX5A (WOX7) | AT5G05770 | Zm_WOX13A | AM234776 |
| At_WOX5B (WOX5) | AT3G11260 | Zm_WOX13B | AM234777 |
| At_WOX6 | AT2G01500 | Zm_WOX13C(ZmWOX14B) | EU952747 |
| At_WOX8 | AY251400 | Zm_WOX13D(ZmWOX14A) | EU961475 |
| At_WOX9A (WOX9) | AT2G33880 | Vv_WUS | AM447418 |
| At_WOX9B (WOX8) | AT5G45980 | Vv_WOX1 | AM439847 |
| At_WOX11 | AT3G03660 | Vv_WOX2 | AM488389 |
| At_WOX12 | AT5G17810 | Vv_WOX3 | AM429035 |
| At_WOX13A(WOX13) | AT4G35550 | Vv_WOX4 | AM447494 |
| At_WOX13B(WOX10) | AT1G20710 | Vv_WOX5 | AM454567 |
| At_WOX14 WOX13C) | AT1G20700 | Vv_WOX6 (VvWOX1B) | AM463144 |
| Gb_WUS | FM882128 | Vv_WOX9 | AM488026 |
| Gb_WOX2 | FM882124 | Vv_WOX11 | AM435207 |
| Gb_WOX3A | FM882125 | Vv_WOX13A | AM486367 |
| Gb_WOX3B | FM882126 | Vv_WOX13B | AM473516 |
| Pp_WOX13-LIKE A | BAM76366 | Vv_WOX13C | AM463736 |
| Pp_WOX13-LIKE B | BAM76367 |  |  |
| Pp_WOX13-LIKE C | BAN16591 |  |  |

**Reference**

Deveaux, Y., Toffano-Nioche, C., Claisse, G., Thareau, V., Morin, H., Laufs, P., et al. (2008). Genes of the most conserved *WOX* clade in plants affect root and flower development in *Arabidopsis*. *BMC Evol Biol* 8**,** 291.

Gambino, G., Minuto, M., Boccacci, P., Perrone, I., Vallania, R., and Gribaudo, I. (2011). Characterization of expression dynamics of WOX homeodomain transcription factors during somatic embryogenesis in *Vitis vinifera*. *J Exp Bot* 62(3)**,** 1089-1101.

Haecker, A., Groß-Hardt, R., Geiges, B., Sarkar, A., Breuninger, H., Herrmann, M., et al. (2004). Expression dynamics of *WOX* genes mark cell fate decisions during early embryonic patterning in *Arabidopsis thaliana*. *Development* 131(3)**,** 657-668.

Hedman, H., Zhu, T., von Arnold, S., and Sohlberg, J.J. (2013). Analysis of the *WUSCHEL-RELATED HOMEOBOX* gene family in the conifer *Picea abies* reveals extensive conservation as well as dynamic patterns. *BMC plant biology* 13(1)**,** 89.
