# Supplementary material for: Desiccation Treatment and Endogenous IAA Levels Are Key Factors Influencing High Frequency Somatic Embryogenesis in Cunninghamia lanceolata (Lamb.) Hook
Source: Front Plant Sci. 2017 Dec 5;8:2054. doi: 10.3389/fpls.2017.02054 (PMC5723420; doi:10.3389/fpls.2017.02054)
Supplement: Supplementary file 4 [file Table_4.DOCX]

Supplementary Material

Desiccation treatment and endogenous IAA levels are key factors influencing high frequency somatic embryogenesis in *Cunninghamia lanceolata* (Lamb.) Hook

Xiaohong Zhou^1,2†^, Renhua Zheng^3†^, Guangxin Liu^1,2^, Yang Xu^1‡^, Yanwei Zhou^1,2^, Thomas Laux^4^, Yan Zhen^1,2^, Scott A. Harding^5^, Jisen Shi^1,2*^, and Jinhui Chen^1,2*^

*** Correspondence:** Dr. Jinhui Chen: Tel.: +86 25 85428817; E-mail: chenjh@njfu.edu.cn; Dr. Jisen Shi: Tel.: +86 25 85428948; Fax: +86 25 85428948; E-mail: jshi@njfu.edu.cn.

## Supplementary Tables

**Supplementary Table S4**. Orthologous contigs of *SOMATIC EMBRYOGENESIS RECEPTOR KINASE* (*SERK*) and *WUSCHEL-related homeobox* (*WOX*) identified by screening the Chinese fir 6421 transcriptome.

|  | **Contig No.** | **Accession No.** | **Reference Sequence Name** | **Gi No.** |
| --- | --- | --- | --- | --- |
| ClWOX4 | 36733, 32906, 32905 | MG431986 | *Picea abies* WOX4 | gi\|498904044 |
| ClWOX5 | 19482 | MG431987 | *Picea abies* WOX5 | gi\|498904046 |
| ClWOX13-1 | 2065 | MG431988 | *Arabidopsis thaliana* WOX8 | gi\|473936590 |
| ClWOX13-2 | 20784, 26020 | MG431989 | *Arabidopsis thaliana* WOX8 | gi\|473936590 |
| ClWOX13-3 | 20417 | MG431990 | *Arabidopsis thaliana* WOX8 | gi\|473936590 |
| ClSERK1-1 | 18030 | MG387961 | *Oryza sativa* Japonica Group SERK2 | gi\|115445577 |
| ClSERK1-2 | 7157 | MG387960 | *Medicago truncatula* SERKL3 | gi\|308154499 |
| ClSERK1-3 | 39103 | MG387964 | *Arabidopsis thaliana* SERK1 | gi\|15217584 |
| ClSERK1-4 | 19371 | MG387962 | *Arabidopsis thaliana* SERK2 | gi\|332193565 |
| ClSERK2-1 | 20090 | MG387963 | *Oryza granulata* SERK1 | gi\|290767960 |
