# Supplementary material for: Desiccation Treatment and Endogenous IAA Levels Are Key Factors Influencing High Frequency Somatic Embryogenesis in Cunninghamia lanceolata (Lamb.) Hook
Source: Front Plant Sci. 2017 Dec 5;8:2054. doi: 10.3389/fpls.2017.02054 (PMC5723420; doi:10.3389/fpls.2017.02054)
Supplement: Supplementary file 5 [file Table_5.DOCX]

Supplementary Material

Desiccation treatment and endogenous IAA levels are key factors influencing high frequency somatic embryogenesis in *Cunninghamia lanceolata* (Lamb.) Hook

Xiaohong Zhou^1,2†^, Renhua Zheng^3†^, Guangxin Liu^1,2^, Yang Xu^1‡^, Yanwei Zhou^1,2^, Thomas Laux^4^, Yan Zhen^1,2^, Scott A. Harding^5^, Jisen Shi^1,2*^, and Jinhui Chen^1,2*^

*** Correspondence:** Dr. Jinhui Chen: Tel.: +86 25 85428817; E-mail: chenjh@njfu.edu.cn; Dr. Jisen Shi: Tel.: +86 25 85428948; Fax: +86 25 85428948; E-mail: jshi@njfu.edu.cn.

## Supplementary Tables

**Supplementary Table S5**. Primer sets used for quantitative RT-PCR.

|  | **Contig No.** | **Primer (5′–3′)** |
| --- | --- | --- |
| ClWOX4 | 36733, 32906, 32905 | F:CCTATCTTCACTTCTTCCTCCTTGTTC  R:TCAATGTCTGTTTTTGTGGGGACC |
| ClWOX5 | 19482 | F:GGTCTGATTTGAATAGCACAGGAGC  R:TTCCCAACAACTGAGAGAGAGGTC |
| ClWOX13-1 | 2065 | F:AAACCCTCTCCAGGAAAGTCAAGC  R:GGCTCATCCACTCTTACTAACTGTCC |
| ClWOX13-2 | 20784, 26020 | F:CAAAACAGGCAACCTACTGCTGACC  R:CATTTCCAGGATTGACACTGACACC |
| ClWOX13-3 | 20417 | F:CCTGTATCAAATCAGCAAGTGTCTGG  R:GAAGTGGGTGAAAAGTAACTCCTGC |
| ClSERK1-1 | 18030 | F:ATTCAGGCGGTTCCAAATCAAAAGT  R:TGTCATCCTCACCAGAAACATCAACA |
| ClSERK1-2 | 7157 | F:CACCTGAACTTGGGAAGATGAGTAG  R:CGCTCCGCTAAGATTGTTGTTTC |
| ClSERK1-3 | 39103 | F:TAGCCCAAAATCCCCCACTACTG  R:AACGCCTGCTTGTTTATCCCTTC |
| ClSERK1-4 | 19371 | F:GCTCAATAATAACAGCCTCTCAGGG  R:CCACTGGACCACAAAGAAGTTGG |
| ClSERK2-1 | 20090 | F:CTCACTCACCCCTTTCGTTCAC  R:GCCACCATAGGAGAAGTCCAATG |
