# Supplementary material for: Desiccation Treatment and Endogenous IAA Levels Are Key Factors Influencing High Frequency Somatic Embryogenesis in Cunninghamia lanceolata (Lamb.) Hook
Source: Front Plant Sci. 2017 Dec 5;8:2054. doi: 10.3389/fpls.2017.02054 (PMC5723420; doi:10.3389/fpls.2017.02054)
Supplement: Supplementary file 6 [file Table_6.DOCX]

Supplementary Material

Desiccation treatment and endogenous IAA levels are key factors influencing high frequency somatic embryogenesis in *Cunninghamia lanceolata* (Lamb.) Hook

Xiaohong Zhou^1,2†^, Renhua Zheng^3†^, Guangxin Liu^1,2^, Yang Xu^1‡^, Yanwei Zhou^1,2^, Thomas Laux^4^, Yan Zhen^1,2^, Scott A. Harding^5^, Jisen Shi^1,2*^, and Jinhui Chen^1,2*^

*** Correspondence:** Dr. Jinhui Chen: Tel.: +86 25 85428817; E-mail: chenjh@njfu.edu.cn; Dr. Jisen Shi: Tel.: +86 25 85428948; Fax: +86 25 85428948; E-mail: jshi@njfu.edu.cn.

## Supplementary Tables

**Supplementary Table S6.** The relative lengths and ratios of the chromosomal arms of plants regenerated through somatic embryogenesis.

| **No. of chromosome** | **SL^a^** | **LL^b^** | **L/S^c^** |
| --- | --- | --- | --- |
| 1 | 6.548 ± 0.075 | 7.366 ± 0.069 | 1.125 |
| 2 | 5.758 ± 0.093 | 6.608 ± 0.173 | 1.148 |
| 3 | 5.288 ± 0.119 | 5.792 ± 0.112 | 1.095 |
| 4 | 4.543 ± 0.075 | 6.265 ± 0.050 | 1.379 |
| 5 | 4.154 ± 0.088 | 4.800 ± 0.099 | 1.156 |
| 6 | 3.681 ± 0.048 | 4.682 ± 0.105 | 1.272 |
| 7 | 3.275 ± 0.061 | 4.671 ± 0.047 | 1.426 |
| 8 | 3.525 ± 0.011 | 4.294 ± 0.013 | 1.218 |
| 9 | 3.175 ± 0.049 | 3.936 ± 0.012 | 1.240 |
| 10 | 2.780 ± 0.075 | 3.503 ± 0.012 | 1.260 |
| 11 | 2.518 ± 0.016 | 3.112 ± 0.018 | 1.238 |

^a^SL = (Length of the shorter arm of each chromosome /Sum length of all chromosomes) ×100 ± Standard deviation;

^b^LL = (Length of the longer arm of each chromosome/Sum length of all chromosomes) × 100 ± Standard deviation;

^c^L/S = length of the longer arm/the length of the shorter arm.
