# Supplementary material for: Desiccation Treatment and Endogenous IAA Levels Are Key Factors Influencing High Frequency Somatic Embryogenesis in Cunninghamia lanceolata (Lamb.) Hook
Source: Front Plant Sci. 2017 Dec 5;8:2054. doi: 10.3389/fpls.2017.02054 (PMC5723420; doi:10.3389/fpls.2017.02054)
Supplement: Supplementary file 8 [file Image_2.PDF]

Supplementary Material

Desiccation treatment and endogenous IAA levels are key factors influencing high frequency somatic embryogenesis in *Cunninghamia lanceolata* (Lamb.) Hook

Xiaohong Zhou[1,2†], Renhua Zheng[3†], Guangxin Liu[1,2], Yang Xu[1‡], Yanwei Zhou[1,2], Thomas Laux[4], Yan Zhen[1,2], Scott A. Harding[5], Jisen Shi[1,2*], Jinhui Chen[1,2*]

*** Correspondence:** Dr. Jinhui Chen: Tel.: +86 25 85428817; E-mail: chenjh@njfu.edu.cn; Dr. Jisen Shi: Tel.: +86 25 85428948; Fax: +86 25 85428948; E-mail: jshi@njfu.edu.cn.

Supplementary Figures

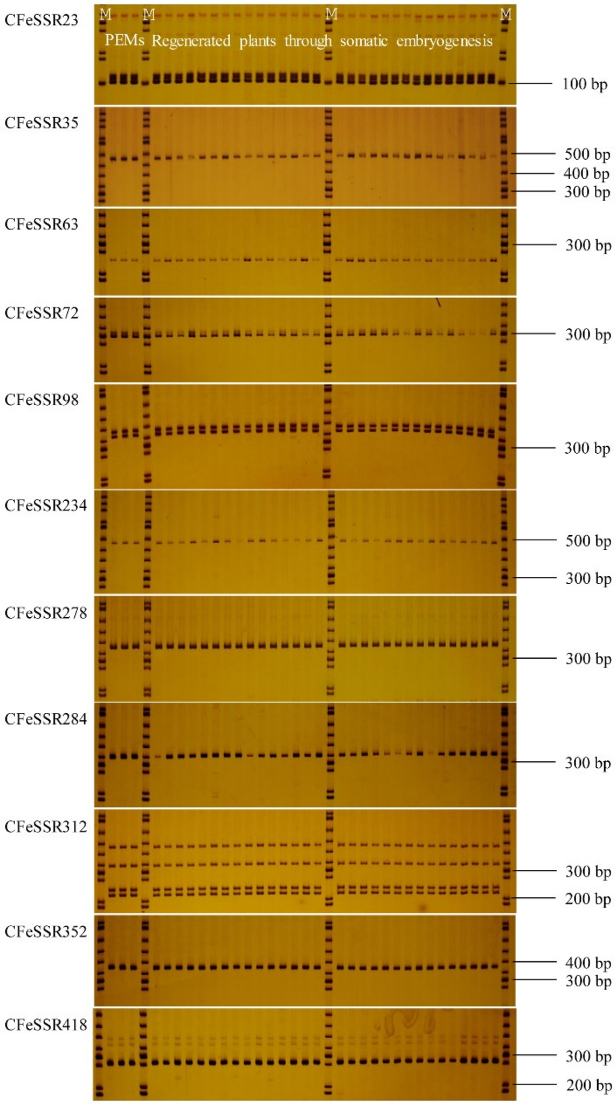

Supplementary Figure 2. SSR analysis of 3 lines of proembryogenic masses (PEMs) and 30 plants (genotype 4098) regenerated via SE using 11 loci. No variation was detected. M = 50-bp ladder; from left to right, the first three samples are PEM lines, followed by the 30 somatic plant samples.
